# A Brief Review on the Analysis of dsDNA, RNA, Amino Acids and Bacteria by Capillary Electrophoresis

**DOI:** 10.3390/bioengineering12121306

**Published:** 2025-11-28

**Authors:** Yuan Zeng, Ping Wang, Bo Yang, Yuqing Xu, Yueqing Wang, Zhenqing Li, Yoshinori Yamaguchi

**Affiliations:** 1 College of Medical Imaging, Shanghai University of Medicine & Health Sciences, Shanghai 201318, China; 2 Department of Clinical Laboratory, Xinhua Hospital, Shanghai Jiao Tong University School of Medicine, 1665 Kongjiang Road, Shanghai 200092, China; 3 Engineering Research Center of Optical Instrument and System, Ministry of Education, Shanghai Key Lab of Modern Optical System, University of Shanghai for Science and Technology, No. 516 JunGong Road, Shanghai 200093, China; 4 Comprehensive Research Organization, Waseda University, Tokyo 162-0041, Japan

**Keywords:** capillary electrophoresis, dsDNA, RNA, amino acid, bacteria, microchip capillary electrophoresis, capillary coating

## Abstract

Capillary electrophoresis (CE) is an effective tool for the analysis of many biocomponents, such as dsDNA, RNA, amino acids and bacteria, which are extremely important not only in research work but also in numerous practical applications. However, there are many factors that affect the separation performance, including the polymers inside the capillary, the electric field strength, the capillary coating and the effective length of the capillary. So far, various CE techniques have been developed to increase the resolution, sample volume consumption and limit of detection. To better understand the development of techniques for the separation of these biomolecules by CE, this review provides a comprehensive summary of polymers (e.g., polyvinylpyrrolidone, hydroxyethyl cellulose and polyethylene glycol), optimization methods, capillary coating methods, technological advancement of microchips for CE and the limitation of detection proposed by different groups worldwide. We also discuss the challenges and future directions associated with CE technology.

## 1. Introduction

Slab-gel electrophoresis is a traditional method for the analysis of dsDNA, RNA and proteins, but it suffers from low throughput, slow analysis and limited resolution, making it challenging to separate closely sized nucleic acids or proteins. It also requires large sample volumes, is labor-intensive and shows poor reproducibility, limiting its suitability for high-throughput or quantitative applications compared with capillary electrophoresis (CE) due to its relatively reduced sample consumption, high resolution, and low limit of detection [1,2,3], making it widely employed in the field of biological and chemical analysis, including for DNA sequencing, protein characterization, drug purity testing, metabolite profiling, and forensic identification [4,5,6].

The development of CE technology has witnessed a continuous process of innovation and improvement. Since its first application in biomolecular analysis in the late 1980s, CE has made significant progress in instrumentation, separation modes, and detection methods [7,8,9,10]. Early research focused on exploring the principles of the technology and optimizing basic separation conditions, gradually expanding into a broader field of biomolecular analysis. For example, Scriba’s group developed and optimized a CE method for determining the enantiomeric purity of silodosin using a Quality-by-Design (QbD) approach. Through factorial and response surface designs, the method was validated and proven robust for both raw materials and pharmaceutical formulations [6]. Jiang et al. successfully detected 13 respiratory pathogens by combining multiplex PCR and CE, providing a rapid and reliable means for clinical microbiological detection [11]. Cottet’s lab presented a quantitative method to evaluate protein adsorption on capillary surfaces in CE by analyzing separation efficiency at varying voltages. It enables fair comparison of coating performance, optimization of electrophoretic conditions, and significant improvement of separation efficiency for protein analysis [12]. Lu developed a capillary gel electrophoresis (CGE) method for high-resolution separation of large RNAs (~2000 nt) under strongly denaturing, non-aqueous conditions using formamide-based gels containing high-molecular-weight polymers. Compared with conventional aqueous CGE, the optimized method improved resolution by about sixfold for RNA ladders of 1500 nt and 2000 nt, demonstrating its strong potential for accurate purity and size analysis of mRNAs in drug and vaccine development [13]. Song’s lab established a rapid and sensitive detection system for four foodborne pathogens by integrating magnetic separation, multiplex PCR (MPCR), and CE. The method achieved a DNA detection limit of 10^−5^–10^−7^ ng μL^−1^ and detected bacterial loads as low as 10 CFU mL^−1^ in food samples, offering higher sensitivity and faster analysis than conventional enrichment-based techniques [14]. Horka achieved online concentration and separation of bacteria by adsorbing bacterial cells on the rough surface of the capillary and utilizing transient isotachophoresis and micellar electrokinetic chromatography techniques [15].

This review summarizes the applications of CE technology in key areas such as biomolecular analysis, microbial detection, and disease diagnosis. It further explores the integration of CE with other analytical techniques, as well as the challenges and future directions in this field. Through an in-depth discussion of these aspects, the review aims to comprehensively highlight the significance and broad application prospects of CE technology in biological analysis, providing valuable insights for researchers and practitioners in related disciplines.

## 2. Main

### 2.1. Analysis of dsDNA by CE

#### 2.1.1. Factors Affecting Separation Performance

Several factors influence DNA analysis in CE. As the concentration of screening polymers increases, the migration time lengthens and mobility decreases, indicating that the degree of entanglement between DNA samples and polymers is concentration-dependent [16]. The radius and viscosity of the polymers are also affected by their hydrophobicity. Hydrophilic polymers tend to form more robust entangled networks, resulting in fewer DNA–polymer hydrophobic interactions, higher resolution, and longer read lengths. However, these polymers exhibit high zero-shear viscosity, requiring increased pressure for loading into microchannels. In contrast, hydrophobic polymer networks are more easily disrupted by DNA migration, leading to reduced resolution and shorter read lengths, but they facilitate easier solution loading due to their lower shear viscosity [17]. Additionally, temperature fluctuations can impact DNA sequencing via CE. The viscosity of the running buffer is influenced by the capillary temperature, which, in turn, it affects electro-osmotic flow (EOF), electrophoretic mobility, and injection volume, thereby affect the separation efficiency [18].

#### 2.1.2. Application of dsDNA-Based CE

CE has numerous applications in DNA research. When drug molecules bind to DNA, they alter the DNA’s charge and shape, which, in turn, affect its migration speed. By examining the binding behavior of ibuprofen with three cyclodextrin species using electrospray ionization mass spectrometry (ESI-MS) and affinity CE (ACE), notable differences in binding interactions were observed between the gas phase (ESI-MS) and the liquid phase (ACE). Molecular docking and force-field simulations indicate that the absence of water molecules in the gas phase results in altered binding affinities. In the liquid phase, β-CD forms the most stable complex with ibuprofen, whereas γ-CD exhibits weaker binding in both environments. This study underscores the complementary roles of ESI-MS for rapid screening and ACE for detailed binding affinity analysis in drug discovery [19]. Janan et al. developed a CE-based method for analyzing DNA origami nanostructures, highlighting the potential of CE in processing highly structured and diverse DNA nanostructures. This approach enables researchers to evaluate the morphology, size, and purity of DNA origami, providing valuable experimental foundations for nanotechnology and biomedical applications [20]. Additionally, CE plays a crucial role in the quantitative determination of electrochemically induced DNA damage. When soluble nucleic acids interact with a high-surface-area mesh carbon electrode at a constant potential, direct strand cleavage of DNA occurs, allowing for the quantitative analysis of the cleavage products via CE. Exposure of calf thymus DNA, DNA gradients, and two types of 40-mer oligonucleotides to electrode potentials ranging from 0.5 to 3V (vs Ag/AgCl) for 1 h offers a method to simulate an oxidative environment without the need for additional reagents. The combination of electrochemical oxidation and molecular analysis provides a means to measure DNA damage thresholds and assess the sensitivity of specific DNA base pairs to oxidative stress, offering new insights into the mechanisms of oxidative DNA damage [21].

#### 2.1.3. Optimization and Improvement

**Figure 1:**
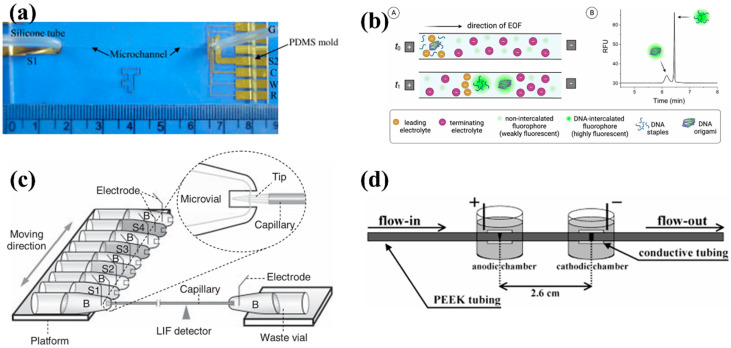
(**a**) Image of a CE-AD microchip showing the microchannel and contact pads (W, working; C, counter; R, reference; G, decoupler ground electrodes; S1 and S2, separation electrodes; AD, amperometric detection) [22]. (**b**) Schematic of capillary electrophoresis (CE) for analysis of DNA origami nanostructures. (**A**) Separation of the nanorod (NR) DNA origami from excess staple strands via CE, employing capillary transient isotachophoresis (ctITP) for analyte focusing. DNA was stained on the column with a noncovalent fluorophore that exhibits intense fluorescence upon intercalation. (**B**) A representative electropherogram demonstrating the high resolution and peak efficiency of the separated analytes (RFU, relative fluorescence units) [23]. (**c**) Schematic diagram (not to scale) of the system for separating short DNA fragments using a short capillary. (**B**) Key components of the separation system (‘S’ denotes the sample inlet, with S1–S4 representing samples 1–4; ‘B’ denotes the buffer reservoir containing the sieving matrix) [26]. (**d**) Schematic of the key fluidic components in the DNA capture device [27].

Research on CE in the field of DNA analysis is progressing steadily. The microchip (Figure 1a) developed by Jang et al. demonstrates excellent performance in terms of sample volume and detection sensitivity. They separated and analyzed small DNA fragments using CE with amperometric detection on inexpensive disposable glass microchips. The microchips featured microchannels carved from polydimethylsiloxane and Au/Ti microelectrodes for sample detection. DNA fragments were separated under a low electric field (20 V/cm) to enhance detection sensitivity while preserving the native conformation of the biomolecules. This method exhibits high reproducibility for small sample volumes (as low as 1 μL) and successfully achieves the separation and detection of DNA fragments of varying lengths [22]. For the analysis of DNA origami (Figure 1b) nanostructures using CE, researchers optimized the separation of folded DNA origami nanostructures from excess staple chains in CZE and ctITP modes. In CZE mode, separation efficiency is improved by optimizing the buffer composition (such as by adjusting Tris, magnesium ion concentration, and pH), selecting appropriate fluorescent dyes (such as SYBR series dyes), and optimizing instrument parameters (e.g., capillary length, voltage, and injection pressure). The ctITP mode utilizes the stacking effect of the sample between electrolytes with different migration rates to focus the analyte peak, further improving the separation performance [23]. We presented a pore-size controllable hydrogel synthesized for CGE, using symmetrical tetrahedron-like macromonomers pentaerythritoltetra (succinimidylcarboxypentyl) polyoxyethylene (PS) and pentaerythritoltetra (aminopropyl) polyoxyethylene) (PA). The hydrogel provides optimal DNA separation when the molecular weights of PA and PS are similar. It successfully resolves DNA fragments under 1500 bp in 13 min and allows for over 100 consecutive runs before performance degradation. Notably, the hydrogel achieves single-base-pair resolution in dsDNA separation, distinguishing even 1 bp differences in length [24]. Wang et al. developed a CE method with fluorescence detection and a partial-filling mode for chiral separation of ofloxacin, using DNA oligonucleotides with different base sequences (tetrahedron, G-quadruplex, and G-rich double strands) as chiral selectors. The optimized method achieved excellent chiral separation, with resolutions above 1.5, and demonstrated repeatability, with relative standard deviations below 4%. The results suggest that both the spatial structure and base arrangement of DNA enhance its chiral separation capability. This work paves the way for using DNA as a chiral selector for enantioseparation [25]. These research outcomes lay a foundation for the further application and development of CE technology in DNA analysis, expanding its use to more fields.

### 2.2. Analysis of RNA by CE

#### 2.2.1. Polymers Employed in the Separation of RNA by CE

In CE, the choice of screening polymers significantly affects RNA separation. Polymers with different molecular weights and concentrations, such as hydroxyethyl cellulose (HEC), polyethylene glycol (PEG), and polyethylene oxide (PEO), exhibit varying separation performances. Low-molecular-weight HEC (90k) is beneficial for the separation of short RNA (<1000 nt), and the resolution improves with increasing polymer concentration. In the case of long-chain HEC (250 k, 720 k, and 1300 k), the resolution of short RNA fragments improves with increasing concentration, while the resolution of large RNA fragments (>3000 nt) deteriorates [28]. The separation performance of small RNA fragments (<1000 nt) improves with increasing concentration of PEG/PEO, while the separation performance of large RNA fragments (>4000 nt) deteriorates when the polymer concentrations exceed 1.0%/0.6%. A double logarithmic plot reveals three migration patterns of RNA in PEG (300–500 kDa) and PEO (4000 kDa). Under optimal conditions, the minimum resolvable nucleotide length for RNA is approximately 6.0 nt. PEO (4000 kDa) shows better separation performance for small RNA, with high concentrations of PEO favoring small RNA and low concentrations of PEO benefiting large RNA [29]. Additionally, the role of pulsed electric fields in RNA separation has garnered attention. Unlike the situation with DNA in PFCE, the pulsed electric field primarily affects the separation performance of RNA fragments ranging from 0.4 to 2.0 knt, while the mobility of long RNA fragments is less influenced by modulation depth and pulse frequency. The logarithm of RNA mobility exhibits an approximately inverse relationship with the logarithm of molecular size within a certain range (correlation coefficient: R > 0.99) [30].

#### 2.2.2. The Application in RNA Research

**Figure 2:**
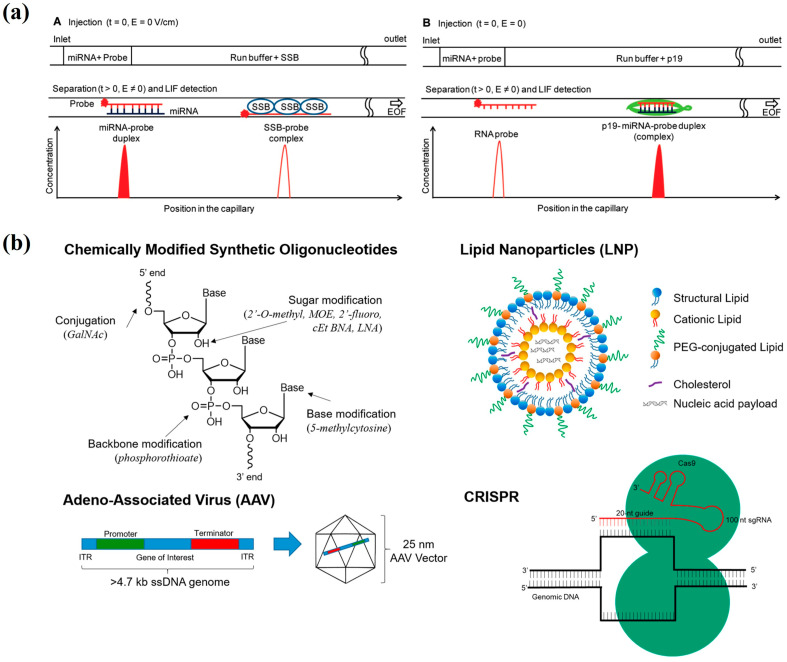
(**a**) Principle of the ProFACE miRNA assay. Following the injection of miRNA and a fluorescent probe, electrophoresis separates the species. (**A**) SSB in the buffer binds the free probe, sharpening the duplex peak. (**B**) p19 in the buffer binds the duplex, shifting its migration and boosting sensitivity [31]. (**b**) Major nucleic acid platforms include chemically modified oligonucleotides, lipid nanoparticles (LNP), adeno-associated virus (AAV) delivery vectors, and CRISPR-based gene editing [34].

Nasrin et al. proposed a protein-assisted affinity CE method (Figure 2a) that utilizes single-stranded DNA-binding protein (SSB) and double-stranded RNA-binding protein (p19) as separation enhancers to quantitatively detect miRNA levels in serum, focusing on liver-specific miRNA-122. The limit of detection (LOD) for this method is 0.5 fM, corresponding to 30,000 miRNA molecules in 1 mL of serum. By employing two pre-concentration techniques—magnetic beads coated with p19 for precipitation and CE sample stacking—it can enrich miRNA from serum by more than 1000 times, enabling the detection of low levels of miRNA in serum without PCR amplification [31]. Johnson et al. presented an optimized high-throughput method for quantifying short nucleic acid reaction products using CE with fluorescence detection on DNA sequencing instruments. The method achieves single-base resolution without extensive sample preparation and extends to RNA quantification. A custom computer program was developed to normalize migration time variations between capillaries for automated kinetic analysis. This approach significantly improves throughput, accuracy, and efficiency, offering a reliable tool for analyzing nucleic acid enzymes in various applications [32]. Sheree et al. developed an eight-marker miRNA panel for body fluid identification (BFID) using CE. The panel includes an endogenous reference gene (let-7g) and specific miRNAs for venous blood, menstrual blood, semen, and saliva. The system uses a linear primer approach for multiplexing, enabling the identification of body fluids in degraded forensic samples. The method successfully distinguishes between body fluids while also generating complete STR profiles from co-extracted DNA for human identification [33]. This indicates that mRNA markers, combined with CE technology, can be effectively employed for forensic fluid identification.

#### 2.2.3. Optimization and Improvement

By optimizing experimental conditions, the resolution and analytical efficiency of RNA separation can be improved. Using polyvinylpyrrolidone (PVP) with an average molecular weight of approximately 1.3 million Da as a high-molecular-weight linear polymer, a low-viscosity sieving medium is prepared by mixing it with glycerol, resulting in a 9% increase in RNA ladder resolution, a 400% increase in peak height, and a 12% reduction in analysis time [13]. To solve the problem of the limited resolution of large RNA separation, 100% formamide was selected as the solvent to prepare CGE gel instead of water. Research shows that formamide not only dissolves charged solutes such as nucleic acids and buffers but also has a strong denaturation ability, enabling it to completely denature large RNA and obtain sharp peaks. The concentration and molecular weight of the polymers (e.g., HEC, PAA, and PEO) mainly affect the separation performance, and it was found that high-molecular-weight PEO (>5 MDa) had the best analysis effect on approximately 2000 nt of RNA at low concentrations (about 0.2% *w*/*v*) [35].

### 2.3. Analysis of Amino Acids by CE

#### 2.3.1. Application in Amino Acid Analysis

Free amino acids play an important role in metabolic monitoring in organisms, and CE is widely used in amino acid analysis [36,37]. Marina investigated four amino acid chiral ionic liquids (CILs) combined with hydroxypropyl-β-cyclodextrin for enantioseparation of seven drugs by CE. The use of [TMA][L-Lys] and [TMA][L-Glu] as modifiers in CE was reported for the first time. Synergistic effects were observed, with resolutions ranging from 1.1 to 6.6 for five compounds. Optimal conditions for enantiomeric separation were achieved by varying the buffer concentration, pH, temperature, and voltage [38]. Zhang explored the use of tetraalkylammonium amino acid ionic liquids (TAA-AAILs) as chiral ligands in ligand-exchange CE (LE-CE) for enantioseparation. Tetraalkylammonium-l-arginine (TMA-l-Arg), TMA-l-proline (TMA-l-Pro), and TMA-l-glutamic acid (TMA-l-Glu) were applied, with TMA-l-Arg showing superior or comparable resolutions relative to previously reported ligands. Key parameters, including the IL concentration, metal ion type, buffer pH, and voltage, were optimized. The method was successfully used for the enantiomeric impurity testing of commercial amino acids, highlighting the potential of TAA-AAILs for enantioseparation in LE-CE [39]. Ganzera et al. presented the first CE method for quantifying mycosporine-like amino acids (AAs) in marine species. Using a 30 mM sodium tetraborate buffer at a pH of 10.3, the method achieved baseline separation of five MAAs—palythine, mycosporine-serinol, asterina-330, shinorine, and porphyra-334—in 27 min. The method was validated and applied to determine AAs in marine macroalgae *Palmaria palmata*, *Porphyra umbilicalis*, and *Porphyra* sp. and lichen *Lichina pygmaea*, offering a reliable approach to the study of photo-protective compounds in marine organisms [40]. Tůma developed a microdialysis–capillary electrophoresis method for AA analysis using a custom coaxial probe fabricated from medical-grade polysulfone hollow fibers. The miniaturized design (5 cm in length and a 200 μm inner diameter) enables efficient microdialysis of 10 μL biological samples directly collected in fused silica capillaries. Using 0.01 M HCl as perfusate under stopped-flow conditions, the method achieved 98.3–102.5% recoveries for 11 amino acids at a 100 μM concentration. Separation was completed within 4.5 min using 8.5 M acetic acid (pH of 1.37) as a background electrolyte, with LODs of 0.12–0.28 μM and peak area reproducibility of 1.2–4.5%. The methodology successfully monitored valine and leucine concentration changes in plasma during fasting and recovery [41]. Emmer et al. developed a CE method with contactless conductivity detection (CE-C^4^D) for comprehensive amino acid analysis. By integrating results from two separation methods using acetic acid and cyclodextrin-based background electrolytes, the approach enabled the monitoring of 17 amino acids in CHO cell culture supernatants. Requiring only sample dilution as pretreatment, the methodology successfully tracked dynamic concentration changes during cell cultivation, demonstrating its potential for bioprocess monitoring [42].

#### 2.3.2. Optimization and Improvement

**Figure 3:**
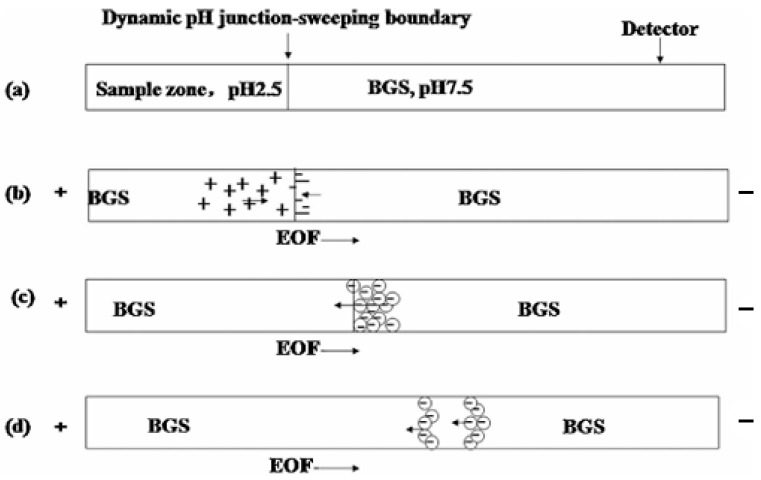
Schematic of the online CE concentration for Glu and Asp using a dynamic pH junction-sweeping technique. (**a**) Capillary conditioning and large-volume sample injection. (**b**) Initiation of the dynamic pH junction. (**c**) Sweeping via analyte-β-CD interaction. (**d**) Final CZE separation [43]. CZE, capillary zone electrophoresis; BGS, background solution; EOF, electro-osmotic flow.

Free amino acids present challenges in CE analysis, prompting many studies to optimize strategies for enhancing accuracy and sensitivity. A method using sodium carbonate buffer and para-amino salicylic acid as the running electrolyte has been successfully applied to the analysis of amino acids in plasma and macrophage culture supernatants (Figure 3). Using benzoic acid as a UV absorption probe and β-cyclodextrin as a scanning agent, the LODs of Glu and Asp were found to be 0.061 and 0.032 μg/mL, respectively, under optimized conditions. The sensitivity was 30–55 times as much as that of traditional CE. The separation of Asp and interfering substances in serum was improved by adding 6% (*v*/*v*) methanol, with serum recovery rates of 82% (Glu) and 87% (Asp) and RSD values of 1.9% and 2.0%, respectively [43]. Na et al. developed an enantiomeric separation method for underivatized free amino acids (AAs) in vinegars using CE coupled with mass spectrometry (CE-MS) and partial filling. Seventeen AAs, excluding proline and asparagine, were successfully separated, with chiral resolution values (Rs) ranging from 0.5 to 21.0. The method utilizes (18-crown-6)-2,3,11,12-tetracarboxylic acid (18C6H4) as the chiral selector, achieving baseline separation for 11 AAs. The optimized method, with detection limits ranging from 0.07 to 1.03 μg/mL, was applied to determine DL-AAs in vinegars and offers potential for analysis of minor enantiomeric impurities in complex samples [40]. Marques et al. used capillary zone electrophoresis with UV–vis detection and principal component analysis to classify craft beer based on amino acid (AA) content. The monitored AAs—cysteine, histidine, phenylalanine, lysine, tryptophan, and arginine—were extracted from wort and finished beer. The analysis revealed distinct AA profiles for each wort, which can serve as quality indicators. One sample demonstrated increased AA concentrations during the mashing step, reflecting protein cleavage [44]. Willis presented two CE methods for resolving 17 amino acids, including seven enantiomer pairs and three achiral amino acids found in both biotic and abiotic samples. One method uses a background electrolyte with γ-cyclodextrin and sodium taurocholate micelles for neutral amino acids and γ-cyclodextrin alone for acidic amino acids. These methods achieve detection limits of 5 nM for neutral and 500 nM for acidic amino acids and have been applied to analyze samples from Mono Lake with minimal preparation [45].

### 2.4. Analysis of Bacteria by CE

#### 2.4.1. Application in Bacteria

**Figure 4:**
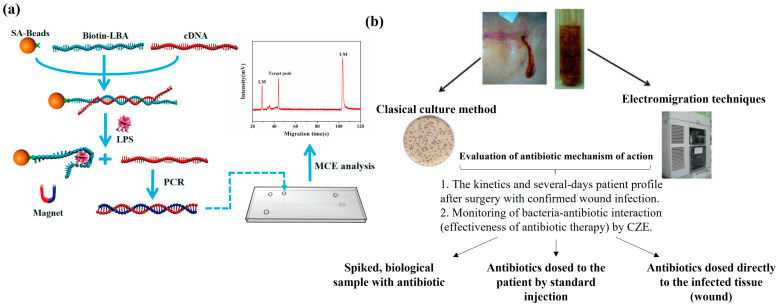
(**a**) Schematic of the MCE strategy coupling aptamer-functionalized magnetic beads with PCR for LPS detection [50]. (**b**) Scheme of the analytical process for the assessment of postoperative infections [46]. LPS, lipopolysaccharide; MCE, microchip electrophoresis.

Bacterial testing is crucial in food safety and medical diagnosis. CE is used to evaluate bacterial–antibiotic interaction in patients with postoperative wound infections and to estimate the effectiveness of antibiotic treatment (Figure 4b), in addition to detecting biological samples at different time points, analyzing changes in bacterial migration time, and determining the optimal conditions for detecting *E. coli* cells. Finally, the optimal migration time was determined to be less than 3.5 min, with sensitivity and specificity of 89.5% and 100%, respectively [46]. It was validated by Guillaume et al. that CE can quickly detect heterogeneous populations of polymyxin-resistant *Klebsiella pneumoniae*. In the future, research will be conducted on other Gram-negative bacteria, with CE expected to be used to monitor changes in bacterial populations in patients treated with polymyxin and study surface chemical-related phenomena of bacteria [47]. Moreover, Marie et al. demonstrated that CZE can quickly and cost effectively detect MRSA strains in blood, distinguish MRSA and MSSA cells in blood culture, and purify enough cells for rapid screening of clinical samples. However, the RSD of the test results fluctuates, and the purification procedure needs to be improved to increase the LOD [48]. The CE technique using the “three-plug injection” method can also quickly detect bacteria in urine samples from patients with urinary tract infection. This involves pretreatment and cleaning of the capillary before the experiment, followed by three injections (sample plug, spacer plug, and blocker plug) during operation and the use of an electro-injection method for injection. This method can detect bacteria with a minimum concentration of 10^6^ CFU/mL within 10 min. If equipped with a laser-induced fluorescence (LIF) detector, the minimum concentration can be reduced to 10^4^ CFU/mL [49].

#### 2.4.2. Optimization and Improvement

The heterogeneity and surface charge of bacteria lead to bacterial aggregation and adsorption issues in CE analysis [51]. To address these issues, modifications can be made to the bacterial cell surface and the inner surface of the capillary (Figure 4a). For example, calcium ion modification can promote bacterial aggregation and reduce signal quantity, and CE can focus on bacteria in isokinetic electrophoresis mode, supporting quantitative analysis [52]. CE analysis of microbial aggregates can also be used to detect and identify microorganisms such as *E. coli*, lactic acid bacteria, and brewing yeast. By surface charge modification, CE can effectively separate microbial aggregates. Combined with MALDI-TOF MS, it can accurately identify bacteria, providing a new method for microbial analysis [53].In addition, Farid et al. used CE technology with multiple UV detection points to evaluate the antibacterial activity of cationic antibacterial compounds against bacteria. They established a new method based on anionic isokinetic electrophoresis and multiple UV detection points to study the lytic activity and adsorption of cationic compounds on bacteria [54].

### 2.5. Methods of Capillary Coating

**Figure 5:**
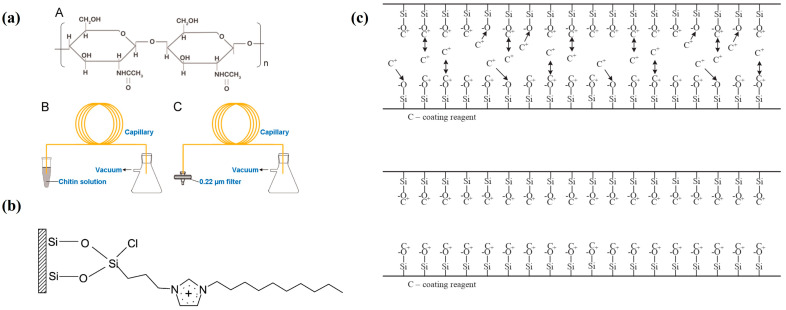
(**a**) Structure of chitin and the coating procedure of a bare capillary [57]. (**b**) Schematic representation of an IL-coated capillary surface [58]. (**c**) Dynamic and physically adsorbed permanent capillary coatings [55].

CE has important applications in the field of bioanalysis, but it faces challenges such as protein adsorption and instability of electro-osmotic flow (EOF) [55]. Novel coatings based on N-acryloylaminoethoxyethanol can withstand harsh conditions, making them suitable for complex sample analysis [56]. Using natural chitin as the material, capillary coatings were prepared by one-step coating method after dissolution with hexafluoroisopropanol (Figure 5a). The coating has excellent stability and can effectively inhibit the adsorption of alkaline proteins on the inner wall of capillaries, achieving rapid and efficient separation of four alkaline proteins. It remained stable during the two-month research period, providing an ideal coating material for protein separation in CE [57]. Qin et al. prepared an ionic liquid (IL)-coated capillary for DNA separation (Figure 5b). Research showed that IL coatings could reverse electro-osmotic flow and have strong interactions with DNA fragments in low-ionic-strength buffer solutions, which depend on fragment charge density. In the presence of HEC, the DNA separation time is shorter than that of polyamide-coated capillaries, and the coating stability is excellent, so it can work stably in running buffer for at least 96 h [58]. To address these issues, researchers have explored various novel coating materials (Figure 5c). The bacterial surface-layer protein (such as SlpA) coating exhibits high stability and excellent performance under high-pH conditions, effectively separating serum lipoproteins [59]. Yu et al. developed a capillary coating system by modifying vancomycin onto the capillary inner wall using photosensitive diazo resin (DR). The coated capillary demonstrates both anti-protein adsorption and chiral separation properties, effectively separating proteins and chiral drugs. It addressed issues of low separation efficiency and high sample loss, offering insights for future coated capillary development [60]. Hara et al. explored the development of a CGE system for point-of-care testing (POCT) by optimizing a short-fused silica capillary coated with an acrylamide/acrylic acid copolymer (poly(AM-co-AA)) for DNA separation. By adjusting the acrylic acid content, electro-osmotic flow (EOF) was controlled, enhancing DNA separation. A content of 2.75 mol % AA was optimal for separating DNA ladder samples ≥600 bp, demonstrating the potential of EOF control for short capillaries in POCT applications [61]. Different inner coatings (such as PVA and PDMA) have varying effects on DNA separation. PVA-coated capillaries were found to have high separation efficiency and resolution at all tested HEC concentrations. The PDMA coating has a poor capillary peak shape and separation effect, which may be due to it small inner diameter or poor compatibility with HEC. These novel coating materials provide effective ways to enhance the performance of CE in bioanalysis, with potential applications in biopharmaceuticals and proteomics. Koval et al. described a sensitive CE method for determining three zwitterionic antiepileptics in human serum using electrophoretic stacking. A PAMAPTAC-coated capillary generated reversed electro-osmotic flow in acetic acid electrolyte. High-volume injection enabled analyte focusing with 78-fold sensitivity enhancement. The validated method showed quantification limits of 18.3–22.8 nmol/L and precision under 2.4% for serum samples diluted with acetonitrile [62]. CE with tunable counter-current flow was also developed for the monitoring of ketamine and metabolites in rat serum. PAMAPTAC-coated capillaries (0–6% APTAC) provided electro-osmotic flow control (0–20 × 10^−9^ m^2^V^−1^s^−1^), achieving a resolution of 3.0 for ketamine/norketamine. After acetonitrile deproteinization, samples were analyzed in 500 mM acetic acid, with detection limits of 2.2–4.1 ng/mL and precision <3.3% RSD. The method successfully tracked serum levels after low-dose ketamine administration [63]. PAMAMPS has also been widely adopted as a capillary coating in the literature [64,65,66].

### 2.6. Chip Capillary Electrophoresis

#### 2.6.1. Application in Biological Sample Detection

**Figure 6:**
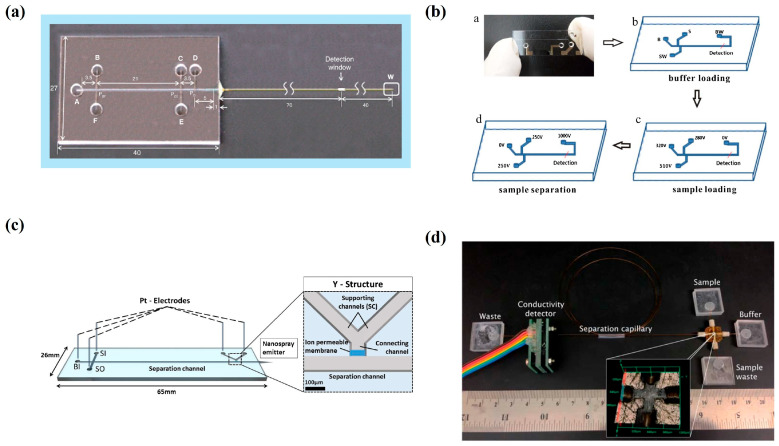
(**a**) An integrated microfluidic chip for protein assay in urine combining t-isotachophoresis with capillary zone electrophoresis [71]. (**b**) Scheme of DNA analysis by MCE: (**a**) microchip; (**b**) buffer loading; (**c**) sample loading; (**d**) separation. Key: S, Sample; SW, Sample Waste; B, Buffer; BW, Buffer Waste [72]. (**c**) Schematic of the integrated CE-MS chip. Electrical contact between the separation channel and nano-spray emitter is achieved through a Y-shaped interface with a conductive membrane, while sample injection is performed via a cross injector [73]. (**d**) Diagram of the 5^2^ platform, comprising five squares joined by capillaries. Inset: micrograph of the central interconnect, measuring 1.28 mm × 1.28 mm. [74].

Chip electrophoresis technology has made significant progress in the analysis of proteins and peptides, continuously innovating in sample preparation [67]. The surface treatment techniques for microchannels are becoming increasingly diverse, and each electrophoretic separation mode each has its own advantages. Detection methods are also continuously evolving, demonstrating broad application prospects in multiple fields [68,69]. A technique based on bacterially specific aptamers combined with microchip CE-LIF has also been developed for the rapid and sensitive detection of *E. coli* in food samples. The LOD is 3.7 × 10^2^ CFU mL^−1^, and the relative standard deviation (RSD) is 0.97% [70]. In urine protein determination, an MC-CE device achieves a lower LOD (TRF of 0.3mg/L, HSA of 0.05mg/L, β 2-MG of 0.6mg/L, and IgG of 0.5mg/L) through sample pretreatment and enrichment on the chip (Figure 6a). The recovery rate is nearly close to 100%. The total analysis time is less than 10 min, making it faster and simpler than a traditional clinical radioimmunoassay. The protein can be baseline-separated after salt removal on the chip and sample purification [71]. Furthermore, Zhang et al. developed a method (Figure 6b) based on polymerase chain reaction (PCR) combined with microchip CE (MCE) for simultaneous detection of three foodborne pathogens (*E. coli*, *Staphylococcus aureus*, and *Salmonella typhimurium*) in food samples. Under optimized conditions, the PCR products of the three pathogenic bacteria were effectively isolated within 135 s, with LOD values (S/N = 3) of 1.6 (*E. coli*), 2.7 (*Staphylococcus aureus*), and 3.5 (*Salmonella typhimurium*) ng/μL. The minimum detectable bacterial counts were 45 (*E. coli*), 62 (*Staphylococcus aureus*), and 42 (*Salmonella typhimurium*) CFU/mL. The RSD values of the peak area and migration time were 0.5–0.7% and 0.5–0.8%, respectively, indicating its high repeatability and precision. The calibration peak height showed a good linear relationship with concentration (R > 0.9985) [72].

#### 2.6.2. Related Equipment and Technological Improvements

Chip electrophoresis-related equipment continues to emerge, and technology is constantly improving. For instance, Scholl et al. developed a new electrical connection method (Figure 6c) by setting an ion-permeable hydrogel membrane in the Y-shaped microfluidic support channel at the end of the separation channel, which avoided problems such as gas and liquid flow interference, as well as sample loss and dilution [73]. A novel mixing platform (Figure 6d) based on five plastic microfluidic components for CE analysis that improves the separation efficiency of sugars was also proposed in [74]. Dou et al. developed a chip CE system with a space-domain internal standard (SDIS) method for rapid detection of *Staphylococcus aureus* (*S. aureus*). The SDIS method enhanced precision and reliability compared to traditional methods. PCR products of the S. aureus nuc gene were quantified in 80 s, with a detection limit of 0.066 ng/μL, offering a promising tool for pathogen analysis [75]. Khatri et al. introduced a microfluid-based CE mass spectrometry (CE-MS) system. By analyzing N-glycans, glycopeptides, and monosaccharides, the sugar-type separation efficiency has improved, and effective separation of glycopeptides and accurate analysis of monosaccharides have been achieved [76].These research results are of great value in promoting progress in this field.

## 3. Conclusions

This review comprehensively summarizes seven key aspects related to CE. Firstly, this article elaborates on how DNA and RNA can be accurately separated and quantified using CE technology, opening up new avenues for gene-related research and deepening molecule-level exploration. At the level of bacterial analysis, the ability of CE to quickly identify bacterial species has greatly improved diagnostic efficiency and environmental monitoring accuracy in the field of microbiology. Amino acid research has emerged in the field of proteomics with the help of this technology, providing powerful tools for analyzing protein structures and exploring the potential of biopharmaceuticals.

The coating method, as a key link in optimizing the CE hardware, effectively overcomes technical obstacles such as electro-osmotic flow and adsorption by improving the surface properties of the capillary, ensuring efficient and stable operation of the experiment. Chip CE, with its advantage of micro-integration, offers innovation with respect to traditional detection modes. It not only significantly reduces sample and reagent consumption but also significantly shortens analysis time, perfectly adapts to real-time detection needs, and empowers medical emergency and on-site rapid testing scenarios.

However, there are still some challenges that need to be overcome in CE technology. In complex sample analysis, matrix interference remains a prominent issue, often leading to limited detection sensitivity and difficulty in meeting the precise determination requirements of trace substances. The relatively high cost of instruments and equipment limits their promotion and popularization in some grassroots laboratories and resource-scarce areas. In addition, the standardization of methods between different laboratories is poor, and the comparability of data needs to be improved, as such barriers hinder the collaborative development and large-scale application of technology. We are eager to see how to overcome the above challenges to apply CE technology more widely.

## Data Availability

No new data were created or analyzed in this study.
